# Hypo-osmotic stress induces the epithelial alarmin IL-33 in the colonic barrier of ulcerative colitis

**DOI:** 10.1038/s41598-022-15573-0

**Published:** 2022-07-07

**Authors:** Mona Dixon Gundersen, Kenneth Bowitz Larsen, Kay Martin Johnsen, Rasmus Goll, Jon Florholmen, Guttorm Haraldsen

**Affiliations:** 1 Research Group of Gastroenterology and Nutrition, Institute of Clinical Medicine, UiT-The Arctic University of Norway, Tromsø, Norway; 2 Department of Medical Biology, Advanced Microscopy Core Facility, UiT-The Arctic University of Norway, Tromsø, Norway; 3 Division of Internal Medicine, Department of Gastroenterology, University Hospital of North Norway, Tromsø, Norway; 4 K.G. Jebsen Inflammation Research Centre, Institute of Clinical Medicine, Faculty of Medicine, University of Oslo, Oslo, Norway

**Keywords:** Cytokines, Inflammatory bowel disease, Mucosal immunology

## Abstract

Epithelial alarmins are gaining interest as therapeutic targets for chronic inflammation. The nuclear alarmin interleukin-33 (IL-33) is upregulated in the colonic mucosa of acute ulcerative colitis (UC) and may represent an early instigator of the inflammatory cascade. However, it is not clear what signals drive the expression of IL-33 in the colonic mucosa, nor is the exact role of IL-33 elucidated. We established an ex vivo model using endoscopic colonic biopsies from healthy controls and UC patients. Colonic biopsies exposed to hypo-osmotic medium induced a strong nuclear IL-33 expression in colonic crypts in both healthy controls and UC biopsies. Mucosal *IL33 *mRNA was also significantly increased following hypo-osmotic stress in healthy controls compared to non-stimulated biopsies (fold change 3.9, p-value < 0.02). We observed a modest induction of IL-33 in response to TGF-beta-1 stimulation, whereas responsiveness to inflammatory cytokines TNF and IFN-gamma was negligible. In conclusion our findings indicate that epithelial IL-33 is induced by hypo-osmotic stress, rather than prototypic proinflammatory cytokines in colonic ex vivo biopsies. This is a novel finding, linking a potent cytokine and alarmin of the innate immune system with cellular stress mechanisms and mucosal inflammation.

## Introduction

The alarmin interleukin-33 (IL-33) belongs to the interleukin-1 family of cytokines and is emerging as an important mediator of epithelial immune responses^1,2^. Alarmins are endogenous molecules that instigate and/or perpetuate inflammation upon release from stressed or damaged cells^3–5^. They swiftly alert the immune system of danger and are gaining interest as upstream targets for chronic inflammatory disease. IL-33 is of particular interest in diseases affecting epithelial borders including asthma, atopic dermatitis and inflammatory bowel disease (IBD)^6–8^. IL-33 is characterised as an intrinsic, nuclear cytokine with a chromatin binding motif. Upon extracellular release, it potently activates innate immune cells expressing the surface membrane bound interleukin-1-like receptor (IL1RL1, alias ST2, IL-33R) resulting in a prompt acute inflammatory response^3,9^. Despite a large body of evidence examining the alarmin functions of IL-33, inducers of IL-33 and its nuclear function are not fully explored and warrant further examination.

Defining mechanisms that induce epithelial IL-33 are of particular interest in ulcerative colitis (UC), one of the main entities of IBD^10^. High IL-33 mucosal levels are a feature of active UC, and it is among the top activated cytokines specific to UC in mucosal gene expression studies ^11^. In addition, polymorphisms of the *IL33 *gene and its ligand receptor *IL1RL1* are associated with an increased risk of UC, implying a role for IL-33 in the disease pathogenesis of UC^12–15^. We and others have reported high accumulation of IL-33 in epithelial crypts during active UC, which is strikingly absent in quiescent disease and healthy controls^13,16,17^. This challenges the view of IL-33 being constitutively expressed in the colonic epithelium and prompts questions into which tissue-specific factors induce synthesis and accumulation of IL-33^13,15,17–19^. Recent reports reveal that interferon gamma (IFN-gamma) and hypo-osmotic stress can induce IL-33 expression in the skin barrier while this remains unknown in gastrointestinal barrier. Osmotic stress is relevant in the pathophysiology of IBD, where loss of intestinal barrier integrity and increased barrier leakiness are well documented^20,21^. Human plasma osmolality is tightly maintained at 277–299 mOsm/kg as fluctuations affect cell volume and function^22^. Exposure to a hypo-osmotic environment results in fluid flux into the cell which will swell^22^. Both hyper- and hypo-osmotic states are described in the colon, influenced by dietary intake, presence of non-absorbable solutes, digestive secretion, (mal)absorption, and intestinal motility^23^. Identifying factors that induce epithelial IL-33 expression may prove beneficial in targeting inflammation in IBD. Moreover, promising clinical trials targeting IL-33 with antibody therapy are reported for chronic inflammatory diseases affecting epithelial borders^8,24^.

There is a pressing need to develop simple and robust human models for examining intestinal mucosal responses^25–27^. This is especially true for IL-33 where a strong interspecies variation has been shown^28^. In this study we established a human colonic ex vivo model based on endoscopy biopsies of healthy controls and patients with UC. IL-33 epithelial expression was investigated with immunostaining and gene mucosal transcription following exposure to the pathophysiological condition of hypo-osmotic stress; proinflammatory mediators interferon gamma (IFN-gamma) and tumour necrosis factor alpha (TNF); and the immune mediator transforming-growth factor beta-1 (TGF-beta-1)^29^.

## Results

### IL-33 expression in treatment naive acute UC

**Figure 1:**
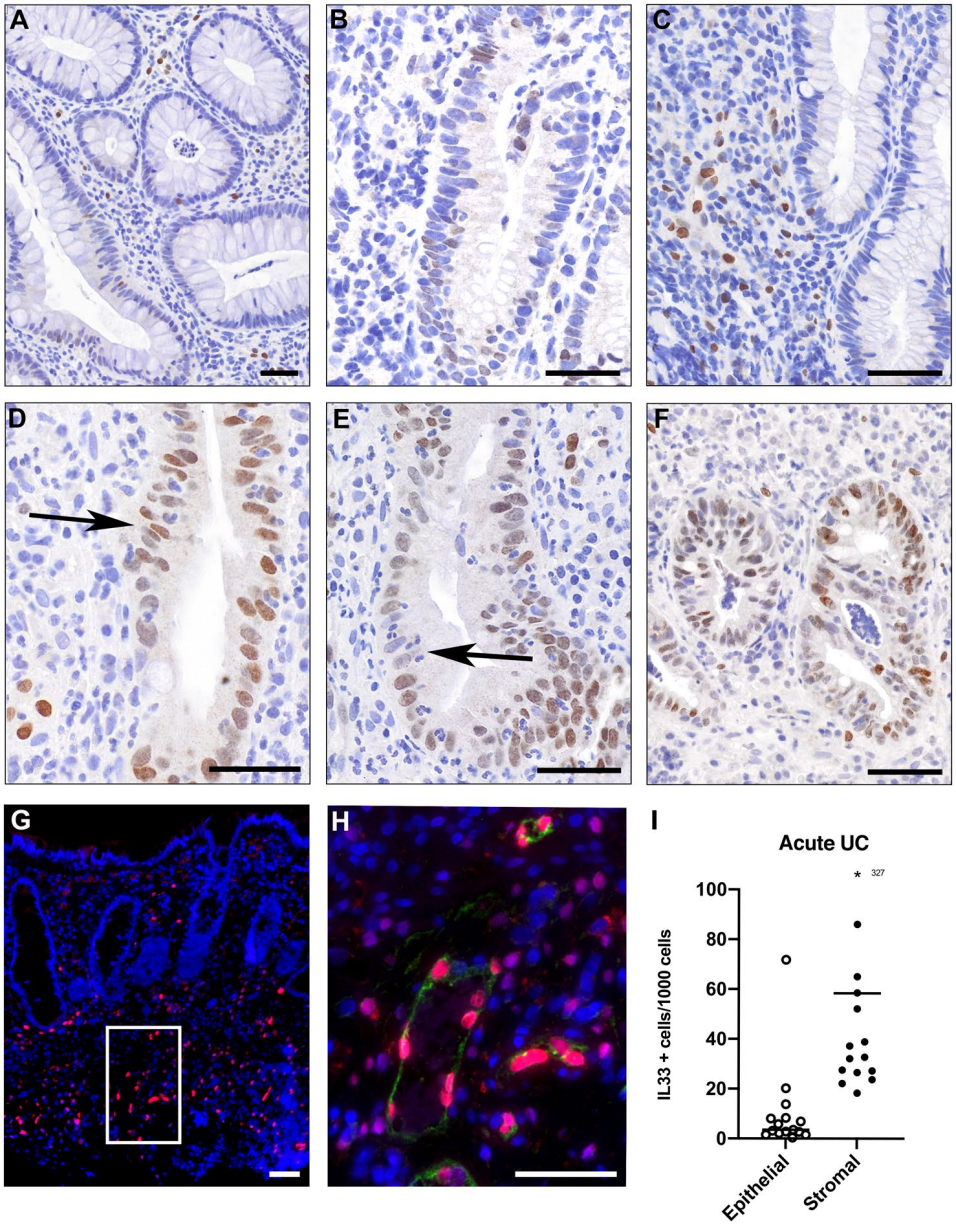
**IL-33 in acute ulcerative colitis (AUC)**. Image (**A-F)** show formalin-fixed, paraffin-embedded colonic biopsies stained for IL-33 (brown) and nuclei stained with hematoxylin (blue). Arrows point to inflammatory cells infiltrating the epithelium. Scales bars = 50 μm. Image (**G)** shows dual staining of AUC with IL-33 (red) and endothelial vessels marked by CD34 (green). White frame in image (**G)** is enlarged as image (**H).** Scale bars = 50 μm. The graph to the far right (**I**) shows positive IL-33 cells per 1000 cells quantified by Qupath analysis software into epithelial and stromal compartments. Asterisk: 327 positive cells/1000 cells.

To determine the distribution of IL-33 in acute UC we examined immunostaining patterns of IL-33 in biopsies from treatment naïve, acute UC patients (n = 15). Patient characteristics are given in Table 1. IL-33 showed a sporadic pattern of IL-33 positive epithelial cells predominately distributed in the mid- and basal parts of colonic crypts in keeping with previously published findings^13,16^. Areas of IL-33 accumulation are shown in Fig. 1. Several epithelial crypts with positive IL-33 expression were associated with the presence of intraepithelial inflammatory cells and crypt-abscesses. We confirmed a strong IL-33 immunosignal in the stroma and dual staining the hematopoietic progenitor cell antigen CD34 confirmed the presence of IL-33 in association with small vessel endothelium in the colon as well in (Fig. 1H).

**Table 1:** Treatment naive acute ulcerative colitis (AUC).

	AUC (n = 15)
Gender (male/female)	7/8
Age median, (range)	35.0 (18–68)
Smoking (never/current/ex-smoker)^a^	5/1/8
Disease distribution (left colitis/extensive)	10/5
Mayo score (median) IQR	7 (6)
Mayo endoscopic subscore (IQR)	2 (0)
Calprotectin (median) (IQR)	975 (1117)
^b^TNF mRNA copies/μg L Median (range) n = 14	11,300 (4600–96,000)

^a^1 unknown.

^b^1 TNF value from baseline characteristics missing.

### Ex vivo model

**Figure 2:**
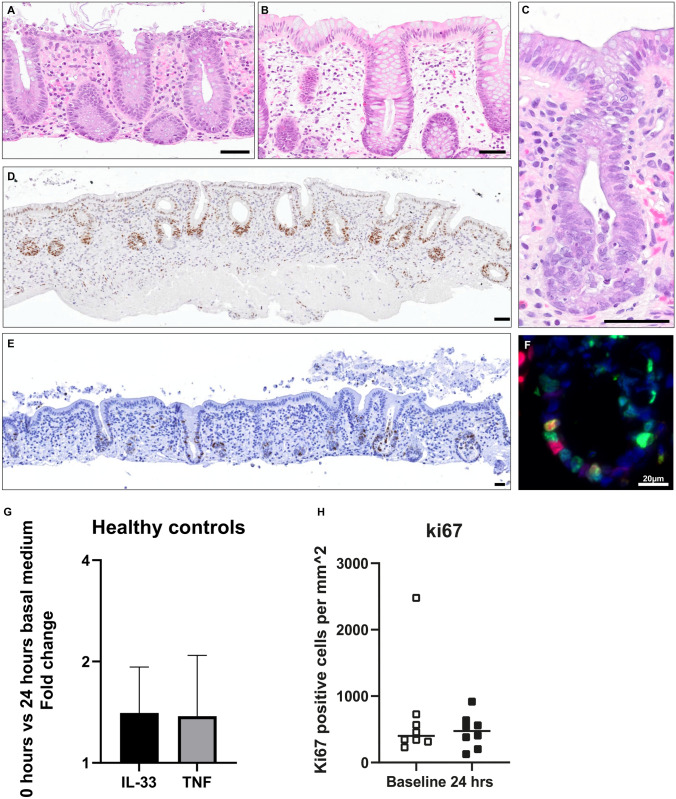
**Ex vivo colonic biopsy model**. Endoscopic pinch biopsies taken from the sigmoid colon were used in an ex vivo biopsy model. Images (**A–C)** show hematoxylin and eosin staining of biopsies from healthy controls following 24 h in basal medium. Image (**D)** shows a biopsy from quiescent UC (Mayo score ≤ 2) stimulated with IFN-gamma 100 ng/ml for 24 h in the basal medium. Immunoenzymatic staining confirms a strong signal of pSTAT1 (brown) in the epithelial border confirming good uptake of IFN-gamma to the epithelium. In image (**E)** the proliferative cell marker Ki67 (brown) was detected with immunostaining in healthy controls at baseline and following 24 h with no significant change in expression shown in graph (**H)**. In image (**F)** dual-staining for IL-33 (red) and Ki67 (green) revealed IL-33 presence also in Ki67 positive cells (yellow). RT-qPCR was performed for *IL33* and *TNF* in healthy controls comparing baseline (0 h) with 24 h incubation in basal medium. No significant differences were seen (*IL33* p-value = 0.3, *TNF* p-value = 0.8) shown in graph **G**. Scale bars = 50 µm.

We established a colonic ex vivo model using endoscopic biopsies from healthy controls and patients with UC (Table 2). Biopsies were cultured for up to twenty-four hours with preserved morphological features and architecture (Fig. 2A–C). Further, uptake of the pro inflammatory cytokine IFN-gamma from the basal medium to the epithelium was confirmed by a strong signal of phosphorylated signal transducer and activator of transcription 1-alpha/beta (pSTAT1) in the epithelium following IFN-gamma stimulation, but not present in non-stimulated controls after 24 h (Fig. 2D). Additional staining for the cell marker Ki67 confirmed presence of cells in different proliferative states (Fig. 2E,F), and no difference when comparing Ki67 immunostaining between available baseline biopsies (n = 8) compared with biopsies cultured for 24 h (Fig. 2H). Paired t-test analysis of mucosal gene expression comparing baseline and 24 h culture showed no significant differences for *IL33* (p-value = 0.3) or *TNF* (p-value = 0.8) (Fig. 2G). RNA integrity was assessed in healthy control biopsies following 24 h of incubation in basal medium (n = 7) with a medium RIN value of 8.6 (range 7.2–9.1) indicating good quality RNA.

**Table 2:** Baseline characteristics of study participants used in ex vivo biopsy model n = 22.

	Control (n = 14)	UC (n = 8)
Gender male/female	9/5	3/5
Age, median (range)	63 (22–82)	43.5 (19–70)
Biopsy location	Sigmoid	Sigmoid
**Mayo score**		
Inactive	0	5
Mild		2
Moderate		1
Severe		0
Calprotectin	NA	25 (25–175)
**Medication**		
5ASA	0	3
Prednisolone	0	0
MTX/AZA	0	2
Anti-TNF	0	6

Continuous variables given as median with range.

### Hypo-osmotic stress induces epithelial IL-33 in an ex vivo model

**Figure 3:**
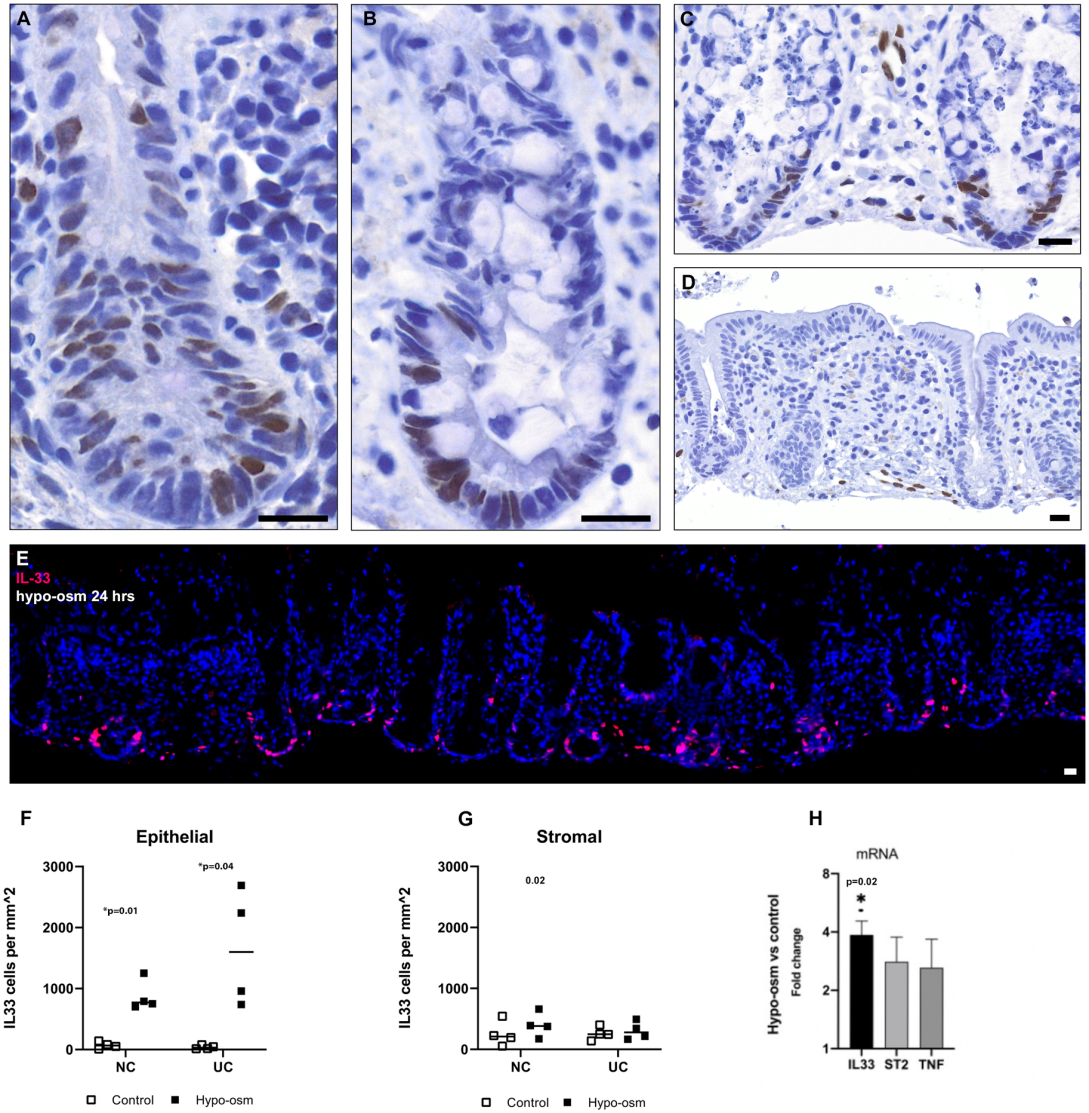
**Hypo-osmotic stress induces IL-33 in colonic crypts**. Colonic biopsies from ex vivo biopsy cultures including quiescent UC with a a Mayo score ≤ 2 (image **A,E**) and healthy controls (image **B–D**). Stimulation with hypo-osmotic medium for 24 h induces positive IL-33 (brown) cells shown with immunoenzymatic staining in (**A–C**). Cell nuclei stained with hematoxylin (blue). Control biopsy cultured for 24 h in basal medium (image **D)** did not show IL-33 positive cells in the epithelium. Image (**E**) shows fluorescence staining in UC after 24 h of hypo-osmotic basal medium, IL-33 (red) is seen in the nucleus of epithelial cells with cell nuclei counterstained with Hoechst (blue). IL-33 was significantly increased in the epithelium of both healthy controls (NC) with a p-value = 0.01 and for UC p-value = 0.04 following hypo-osmotic exposure for 24 h (graph **F**). No significant changes were seen in the stroma (graph **G**) p-values 0.14 and 0.38 for NC and UC. Graph (**H**) shows fold change of mucosal gene expression for *IL33, ST2* and *TNF* comparing biopsies stimulated with hypo-osmotic medium or basal medium (controls) for 24 h. Scale bars = 20 µm.

The colonic epithelial barrier is constantly exposed to osmotic perturbations. In view of the recent report that hypo-osmosis drives IL-33 expression in the skin barrier^30^, we were curious to see whether this also applied to the colonic epithelium. We found that exposure to hypo-osmotic medium (152 mOsm) for 24 h induced a strong nuclear IL-33 signal in the lower part of epithelial crypts in healthy controls (Fig. 3). In contrast, no IL-33 was present in epithelial crypts following 24 h when cultured with regular basal medium (~ 300 mOsm) (Fig. 3D). Further, the majority of IL-33 cells were found to be Ki67 negative, though not exclusively limited to a set cell-proliferative state.

Next, we included colonic biopsies from patients with UC (n = 4) for immunostaining. These patients had an intact mucosal barrier and no presence of ulcers and a Mayo endoscopic score of 0 or 1. We found that hypo-osmotic stress induced epithelial IL-33. The pattern distribution was similar to that observed in mucosal biopsies of acute UC localizing basally in colonic crypts. Mucosal gene expression with qPCR of colonic biopsies showed a significant increase in *IL33* mRNA(fold change 3.9, p value = 0.02) in biopsies exposed to hypo-osmotic stress compared to controls (Fig. 3H). An overview of biopsy stimulation experiments is given in Table 3.

**Table 3:**
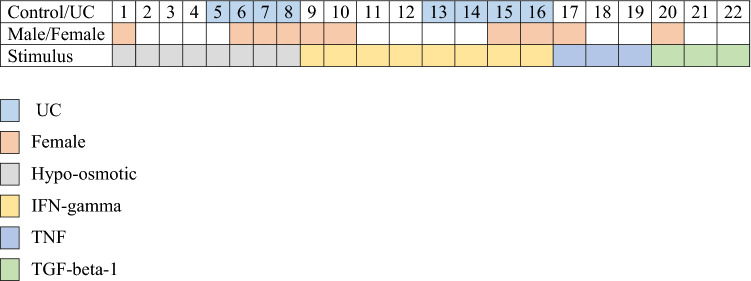
Overview over stimulation experiment setup (n = 22).

### IFN-gamma does not drive epithelial IL-33 in a UC ex vivo model

**Figure 4:**
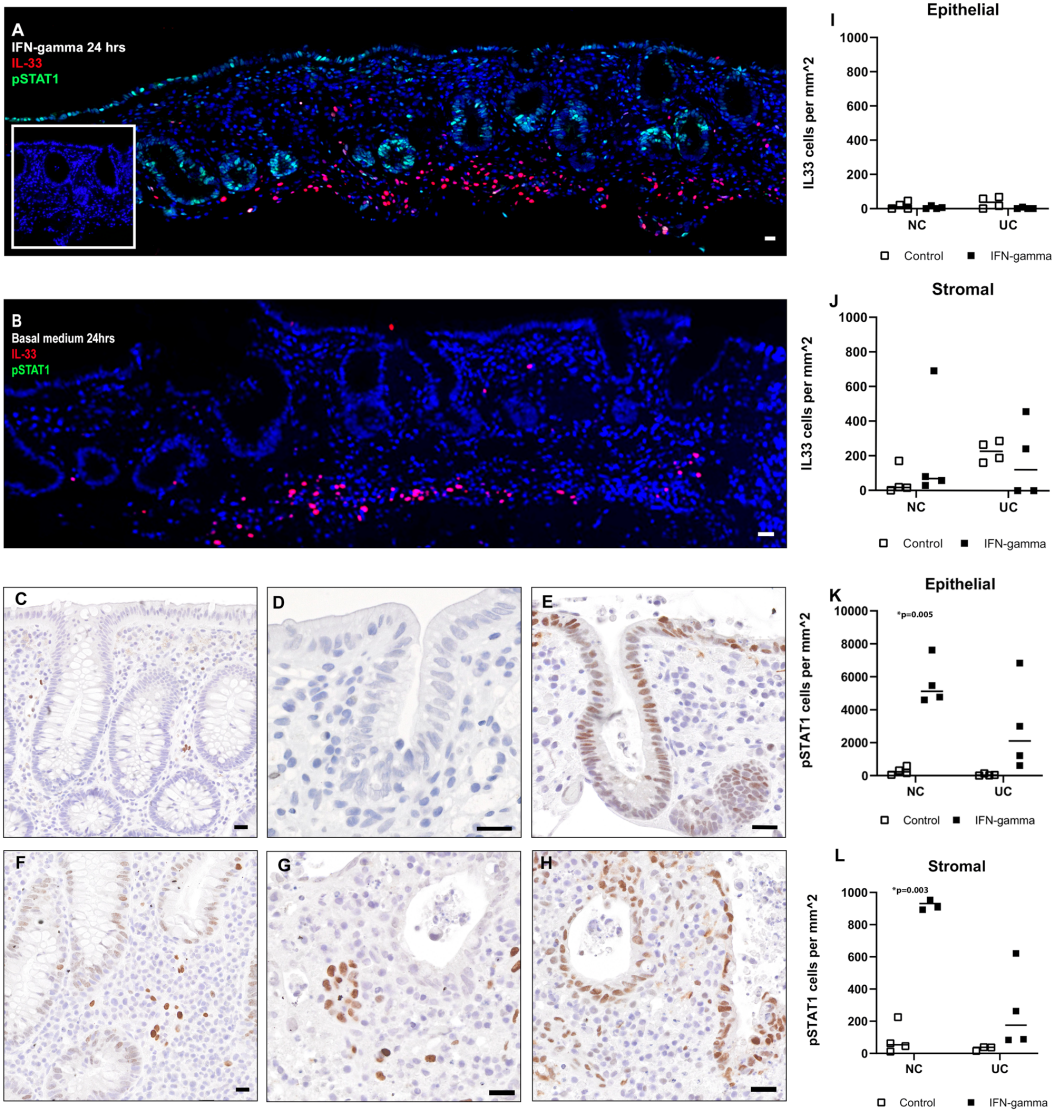
**IFN-gamma stimulation**. Image (**A,B)** show immunofluorescence staining of quiescent UC (Mayo score ≤ 2) in colonic biopsies for IL-33 (red) and pSTAT1 (green) and nuclei counterstained with Hoechst (blue). Image (**A)** shows a strong pSTAT1 signal in epithelial cells following 24 h of IFN-gamma 100 ng/ml stimulation for 24 h. Infelt to the left is an isotype and concentration matched control. Image (**B)** shows the control biopsy cultured in basal medium for 24 h (non-stimulated) with no pSTAT1 epithelial signal. Immunoenzymatic staining for IL-33 (brown) is shown in a healthy control (**C,D)**, Baseline biopsy at 0 h (image **C**) and following 24 h of IFN-gamma stimulation (image **D**) Further pSTAT1 (brown) staining is shown in image **E** after 24 h 100 ng/ml IFN-gamma stimulation. Acute UC (Mayo score = 6) is shown in (**F–H)**. IL-33(brown) is shown in image (**F)** (baseline 0 h) and image (**G)** (stimulated 24 h with IFN-gamma). Image (**H)** shows pSTAT1(brown) following IFN-gamma- stimulation for 24 h. Quantification of immunostaining is given in graphs (**I–L)**. Healthy controls = NC. No significant presence of IL-33 was seen in the epithelium following IFN-gamma stimulation for 24 h. pSTAT1 was significantly increased in the epithelial border and stroma in both healthy controls (NC) and UC. Scale bars are given at 20 μm.

The pro-inflammatory cytokine IFN-gamma has been linked to IL-33 expression in oesophagitis, and is also raised in IBD^31^. IFN-gamma is also a known driver of IL-33 in keratinocytes^28^. We therefore investigated the effect of IFN-gamma on the expression of IL-33 in UC. Inactive UC patients (Mayo endoscopic subscore = 0) did not express IL-33 at baseline, nor following 4 h and 24 h of IFN-gamma stimulation. IL-33 was present in the epithelial at baseline for one patient with moderate UC disease (Mayo endoscopic subscore = 2) but diminished following 24 h of IFN-gamma exposure. In healthy controls (n = 4) we did not observe any significant induction of IL-33 signal in epithelial cells. IFN-gamma at 100 ng/ml was used following 3 log concentration testing (1–100 ng/ml). To ensure adequate IFN-gamma stimulation and uptake throughout the biopsy, we examined expression of pSTAT1, which confirmed a strong epithelial expression following stimulation (Fig. 4).

### TNF did not induce IL-33 in a healthy control ex vivo model

**Figure 5:**
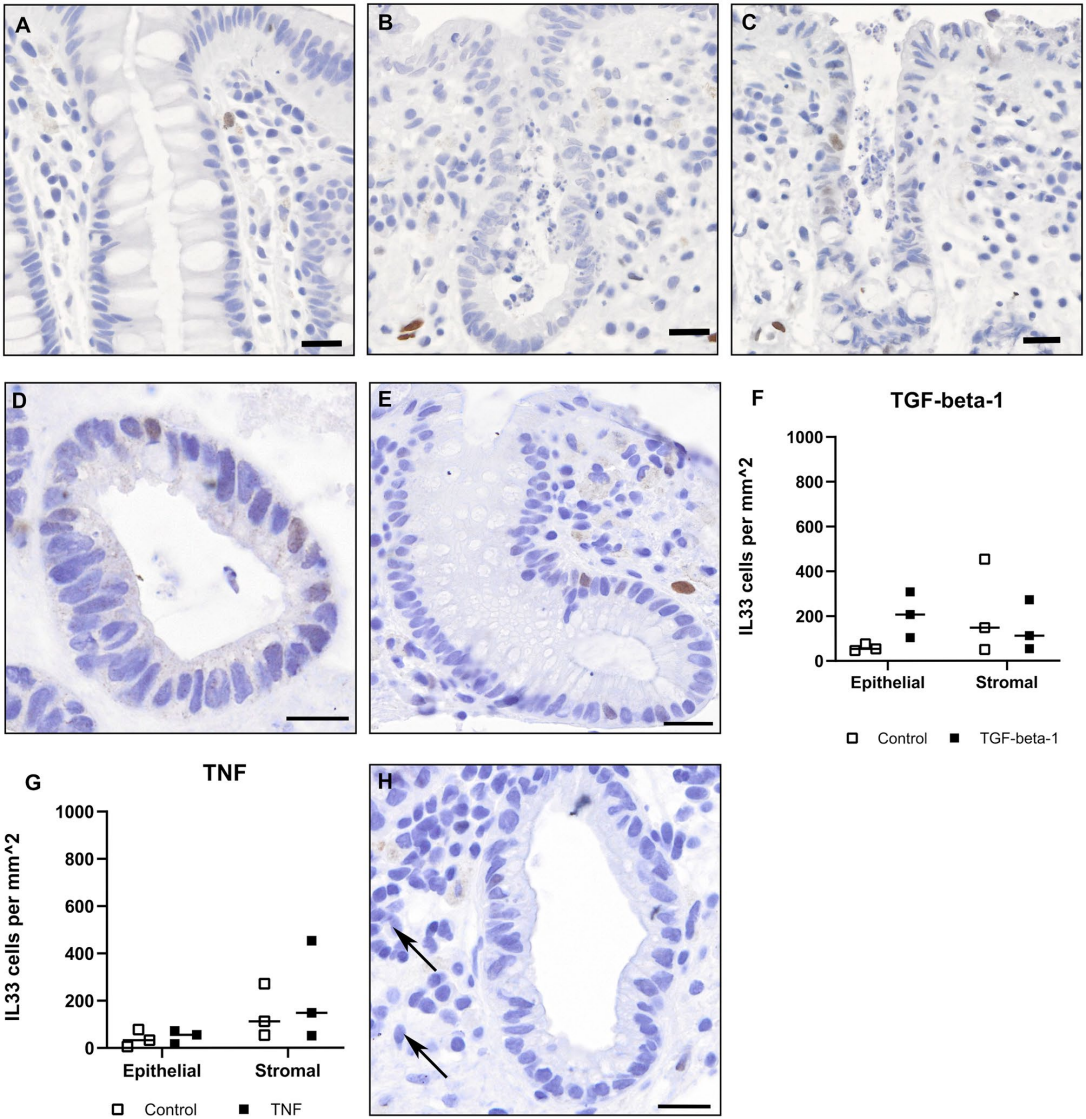
**Ex vivo biopsies stained for IL-33**. Colonic biopsies from healthy controls showing immunoenzymatic staining for IL-33 (brown) and cell nuclei counterstained with hematoxylin (blue). (**A**–**C)** shows biopsies at: baseline 0 hours (**A**) 12 h (**B**) and 24 h (**C**) with stimulation with TGF-beta-1 10 ng/ml. Sporadic but positive epithelial cells for IL-33 were seen. Images (**D,E**) also show healthy controls after 24 h of TGF-beta-1 stimulation. Image (**H**) shows stimulation with proinflammatory cytokine TNF 10 ng/ml for 24 h, with no positive epithelial cells for IL-33, also of note IL-33 in endothelial vessels was lacking (arrows). Scale bars = 20 µm. Graphs (**F**) and (**G**) shown results from image quantification of positive cells.

We also tested TNF and TGF-beta-1 in the range of 3 log concentrations. The pro-inflammatory cytokine TNF is central in IBD and a target for therapy during acute inflammation^29^. IL-33 has also been linked to TGF-beta-1 mediated differentiation of regulatory T-cells in the intestine ^32^. Interestingly, TNF activation (1–100 ng/ml) revealed no evidence of epithelial IL-33 expression, whereas endothelial IL-33 was reduced which is in agreement with reports from in vitro and in vivo studies, supporting the activity of our stimulation experiment^33^. Mucosal biopsies from healthy controls stimulated with TGF-beta-1 at 10 ng/ml for 24 h showed a sporadic but clearly discernible presence of nuclear IL-33 expression in the epithelium and epithelial crypts (see Fig. 5).

## Discussion

In this study we have shown that hypo-osmotic stress induces nuclear IL-33 accumulation in the colonic mucosa in UC and in healthy subjects. This is a novel finding in the colonic mucosa, and links osmotic perturbations of the colonic mucosa with a potent alarmin of the innate immune system.

We observed hypo-osmotic stress to consistently induce IL-33 in both UC and healthy controls. This is in keeping with findings from the skin barrier and may represent a generic mechanism of IL-33 induction. The colonic barrier in IBD is exposed to osmotic stress^21^. This is especially relevant in UC where epithelial barrier dysfunction is as a key feature ^34^. In this pathophysiological context the intestinal barrier may be more sensitive to osmotic perturbations. The literature mainly reports on hyperosmotic stress as a potent inducer of inflammation, acting via NAFT5 inflammasomes and stimulating pro-inflammatory cytokines including IL-8 and TNF^35–38^. Moreover, a recent study found hyperosmotic stress to affect the function of epithelial cells, reducing cell proliferation, mitochondrial function and also altering the gut microbiota^35,39^.Only recently has a hypo-osmotic environment been found to independently instigate inflammation and it remains not well understood^40,41^. It is plausible that disturbance of cell function also occurs following hypo-osmotic stress as seen in reversible cell injury (non-lethal). Mechanisms that respond to cellular swelling include activation of mechanosensitive membrane ion channels such as Piezo1 and osmosensing transient receptor potential vanilloid (TRPV). Aquaporins regulating transcellular fluid flow are also relevant ^42–45^. Our findings support the role of hypo-osmotic stress in inflammation following activation of IL-33 in the epithelial barrier and warrants further exploration. Although the initiating cause of IBD is not clear, identifying the trigger(s) of inflammation are important as different stimuli likely shape and give rise to nuances within the inflammatory response and may aid development of specific therapeutic targets.

In concordance with previous studies, we found IL-33 to accumulate in epithelial crypts of acute UC, whilst consistently absent in healthy controls. This firmly establishes IL-33 as an inducible nuclear cytokine in the healthy colonic barrier^29,46^. Surprisingly, our data did not show the pro-inflammatory cytokines IFN-gamma nor TNF to be of relevance for induction of epithelial IL-33. This is in contrast to findings from squamous cells of the oesophagus and keratinocytes of the skin barrier^28,31^. In support of our findings, we demonstrated a strong pSTAT1 expression in our epithelial colonic border following IFN-gamma stimulation, however we did not have equivalent strong controls for TNF or TGF-beta-1 stimulation. The lack of IL-33 positive endothelial vessels observed following TNF stimulation supports our findings. Moreover, the majority of colonic cell culture studies have not shown stimulation with pro-inflammatory cytokines (TNF, IL1-beta and IFN-gamma) to induce IL-33^14,15,47^. One explanation is that these pro-inflammatory cytokines are unable to induce an epithelial alarmin response alone, perhaps requiring additional mediators activating the inflammasome as seen in mucosal damage driving the generation of reactive oxygen or nitrogen species^48^. Indeed, it remains open if hypo-osmosis activates the expression of enterocyte IL-33 by activation of the inflammasome complex.

By contrast, TGF-beta-1, a pleotropic cytokine with a central role in initiating repair and tissue restoration induced IL-33 in the epithelium albeit less potent than hypo-osmotic stress^49^. IL-33 has been linked to T-cell regulatory mechanisms and proposed a central role in tissue healing following reports from murine models where IL-33 was found important in facilitating wound healing and resolution of inflammation^50^. TGF-beta-1 was also found to induce IL-33 expression in the context of mucosal healing in UC when acting in concert with a TLR3 agonist, like mimicking actions of mRNA released from damaged cells^13^. The idea that alarmins may act both as an inducer and repressor or regulator of the acute inflammatory response is intriguing and underpins the need for further studies to understand the regulation of inflammation to harness therapeutic effects.

The strength of this study includes the use of human colonic biopsies. This is important for any study of IL-33, as strong species differences have been documented^28^. In spite of progress in experimental animal and cell models, important differences may be missed if hypotheses are not tested in human models and correlated to observations in human disease lesions^51^. In this study we show that an ex vivo human biopsy model gives reproducible results to study IL-33 mucosal response and expression. Endoscopic biopsies are routine in the gastroenterology departments, and although not true physiological conditions, the confirmation of IFN-gamma absorbed from the basal medium to the epithelial layer with pSTAT1 supports its use as a powerful, yet mechanistic and simple model that is inexpensive and easy to perform. Advantages to our model include the preserved tissue architecture and cell diversity compared to use of immortal cell lines. The use of antibiotics is arguably a weakness as it inevitably disturbs the gut microbial flora and its interactions with the epithelial lining. Further our experiments are small in sample size, which needs to be taken into consideration when interpreting results. Future perspectives using human ex vivo biopsy models include the possibility to individually tailoring therapy for patients based on cytokine expression and responses.

## Conclusions

In conclusion, hypo-osmotic stress induces a strong pro-inflammatory cytokine in the epithelial colonic border of both healthy controls and active UC. It represents a link between cellular stress and chronic inflammation and may represent a generic IL-33 response in the human body. The use of human ex vivo colonic biopsies performed well as a mechanistic model for studying IL-33.

## Material and methods

### Ethical considerations

The study design, protocol and collection and storage of biopsies was approved by the Regional Committee for Medical and Health Research Ethics, Northern Norway (REK-Nord) (ref: No 2012/1349). The study was conducted according to the Helsinki declaration and all study participants were informed and gave written consent.

### Study participants

Study participants were included from the prospective Advanced Study of IBD (ASIB) at the University Hospital of Northern Norway^52^. Patients with UC were included if 18 years or above. The diagnosis of UC was defined by established clinical criteria (ECCO guidelines), determined by the examining gastroenterologist^53^. Disease activity was assessed using the Mayo clinical score which divides acute UC into categories; inactive (0–2), mild (3–5 points), moderate (6–8 points) and severe (9–12 points). Disease remission was defined as a Mayo score of 2 or less with an endoscopic sub score of 0 and no categories with a score above 1^54^.

### Control group

A normal control group of patients referred for colorectal cancer screening were included if endoscopy was completely normal, confirmed with a normal histology rapport. Exclusion criteria were age < 18 years, history of cancer, irritable bowel disease, chronic inflammation or autoimmune disease in their past medical history.

### Colonic biopsies

#### Ex vivo colon biopsy model

An ex vivo model was established for endoscopic biopsies using a steel mesh to form an air–liquid interface, adapted from principles based on ex vivo models of Brown and Tiering, Vadstrup and Fletcher et al^55–57^. Biopsies were obtained from both healthy controls (as defined above) and patients with UC (including acute and remission states).

Up to four endoscopic biopsies were taken from each patient with a radial jaw forceps (Radial jaw™ 4 3.2 mm, Boston Scientific) from the sigmoid colon, and immediately immersed in Dulbeccos modified Eagle’s medium (DMEM) high glucose (4500 mg/L, l-glutamine, sodium bicarbonate without sodium pyruvate, pH 7.0–7.6. Osmolality 327–361 mOs/kg) (Sigma Aldrich) supplemented with 10% fetal bovine serum with 1% penicillin and streptomycin and gentamycin 50 μg/ml. This transport medium was kept on ice at all times during transport and during orientation of biopsies under a dissecting microscope. Biopsies were placed apical side up on metal grids and placed in a 12-well plate. Basal medium with added glutamine 2 mmol/L with or without stimulants was added to each well up to the metal grid, giving an air–liquid-interface. The ex vivo model was maintained in a humidified atmosphere of 5% C02 and 95% atmospheric air at 37 degrees for maximum 24 h. At experiment end, biopsies were fixated in 10% formalin for 24 h prior to fixation and paraffin embedding. Biopsies for qPCR analysis were fixed in RNA*later*^®^ (Qiagen, Holden, Germany). Biopsy morphology was assessed with hematoxylin & eosin staining (Shandon Instant Hematoxylin and Instant Eosin, Thermo Electron Corporation. Cheshire, UK).

Stimulation with recombinant human IFN-gamma (E.coli derived. Cat.no. 285-IF-100. R&D systems, Minneapolis, MN, USA), rhTNF (E. coli derived.cat.no 210.-TA R&D systems), TGF-beta-1 (HEK293 derived.cat no 7754-BH R&D systems), and hypo-osmotic medium (DMEM basal medium diluted 1:1 with sterile ultrapure H_2_0). All stimulation experiments were repeated three times as a set minimum. Hypo-osmotic medium was measured with FISKE@ microosmometer. model 2/0. (Fiske associates, Massachutes, USA).

To confirm the viability and usefulness of the ex vivo colonic model to our purpose we assessed the expression of pSTAT1 in unstimulated and IFN-gamma-exposed biopsies, observing a strong induction in response to 100 ng/ml IFN-gamma in the epithelial lining of biopsies after 4, 12 and 24 h.

### qPCR analysis

Endoscopic biopsies were obtained with standard forceps, immediately immersed RNA later (Qiagen, Holden, Germany). Biopsies were taken from the sigmoid. RNA was extracted using the AllPrep RNA/DNA miniKit with QIAcube instrument (Qiagen, Hilden, Germany, Cat No:80204) according to manufactures instructions. RNA was stored at −70 °C. Quantity and purity of the extracted RNA was assessed using the Qubit 3 Fluorometer (Invitrogen by Thermo Fisher Scientific, Waltham, MA, USA). QuantiNova reverse transcription Kit (Qiagen, Cat no: 205314) and QuantiNova Probe RT-PR kit (Qiagen Cat no: 208352) were used. Real-time qPCR was performed with Biorad XF96 thermal cycler (Bio-Rad Laboratories AB, Hercules, California, USA) using hydrolysis probes. Forward and reverse primes for *IL33*, *TNF*, *ST2*, *ACTB*, *RPLP0* are previously published^58^. Samples were run in triplicates,The average of reference genes *ACTB* and *RPLP0* were used for normalisation. Data were compared using the delta-delta CT-method and given as fold change^59^.

### Immunostaining

Formalin-fixed, paraffin-embedded samples were cut to 4 μm sections. Slides were rehydrated through a series of xylene and graded alcohol to phosphate buffered saline. Heat induced epitope retrieval was performed in a waterbath for 20 min at boiling temperature with retrieval buffer pH 6 or pH 9 (Dako, Glostrup, Denmark) following 20 min cooling at room temperature. DAKO Envision Flex HRP polymer kit for rabbit/mouse detection was used with 3’3 diaminobenzide using the manual protocol (DAKO, Glostrup, Denmark). Primary antibody incubation for 60 min at room temperature or overnight at 4 °C. DAKO Envision Flex mouse linker was used for primary mouse antibodies (DAKO, Glostrup, Denmark). Counterstain of nuclei with hematoxylin and NaHCO_3_ for nuclear blueing was performed. Sections were dehydrated from alcohol to xylene prior to mounting with Vectamount permanent mounting medium (Vector Laboratories Inc, Burlingame, CA, USA).

Immunofluorescence staining was performed with blocking of section with 10% goat serum for 30 min (Cell signalling Technologies). Primary antibodies were incubated overnight at 4 °C. Secondary goat antibodies conjugated to Alexa 555 or Alexa 647 were used (Life Technologies) and counterstained with Hoechst (33258, Life Technologies). Slides were airdried and mounted with aqueous fluoromount (Sigma-Aldrich). Isotype and concentration matched antibody controls were routinely performed (for antibody details see Supplementary Table S1). Images were captured with VS120 slide scanner (Olympus) for both immunoenzymatic and immunofluorescence staining. Slides were processed using the Olympus OlyVIA 2.9 software. Photos were organised using Adobe photoshop (2020), with adjustment of histograms only made for the whole image.

### Statistics

Mucosal transcripts were analysed using the delta delta CT method for relative quantification, and parametric t-test for independent and paired samples as appropriate were calculated with IBM SPSS statistics 24 (IBM Corporation, Armonk, New York, USA). Fischer’s exact test was used for categorical data. P-values < 0.05 were considered significant. QuPath quantative pathology and bioimage analysis was used for quantification using positive cell detection for both 3’3 diaminobenzide staining and for immunofluorescence staining. The epithelial and stromal compartments were annotated manually^60^.

## Supplementary Information

Supplementary Information.

## Data Availability

The datasets generated during and/or analysed during the current study are available from the corresponding author on reasonable request.

## References

[1] Martin NT, Martin MU (2016). Interleukin 33 is a guardian of barriers and a local alarmin. Nat. Immunol..

[2] Palmer G, Gabay C (2011). Interleukin-33 biology with potential insights into human diseases. Nat. Rev. Rheumatol..

[3] Cayrol C, Girard J-P (2018). Interleukin-33 (IL-33): A nuclear cytokine from the IL-1 family. Immunol. Rev..

[4] Molofsky AB, Savage AK, Locksley RM (2015). Interleukin-33 in tissue homeostasis, injury, and inflammation. Immunity.

[5] Haraldsen G, Balogh J, Pollheimer J, Sponheim J, Kuchler AM (2009). Interleukin-33—Cytokine of dual function or novel alarmin?. Trends Immunol..

[6] Boyapati RK, Rossi AG, Satsangi J, Ho GT (2016). Gut mucosal DAMPs in IBD: from mechanisms to therapeutic implications. Mucosal Immunol..

[7] Bertheloot D, Latz E (2017). HMGB1, IL-1α, IL-33 and S100 proteins: dual-function alarmins. Cell. Mol. Immunol..

[8] Gauvreau GM, White L, Davis BE (2020). Anti-alarmin approaches entering clinical trials. Curr. Opin. Pulm. Med..

[9] Schmitz J (2005). IL-33, an interleukin-1-like cytokine that signals via the IL-1 receptor-related protein ST2 and induces T helper type 2-associated cytokines. Immunity.

[10] Ungaro R, Mehandru S, Allen PB, Peyrin-Biroulet L, Colombel J-F (2017). Ulcerative colitis. Lancet.

[11] West NR (2017). Oncostatin M drives intestinal inflammation and predicts response to tumor necrosis factor-neutralizing therapy in patients with inflammatory bowel disease. Nat. Med..

[12] Latiano A (2013). Associations between genetic polymorphisms in IL-33, IL1R1 and risk for inflammatory bowel disease. PLoS ONE.

[13] Sponheim J (2010). Inflammatory bowel disease-associated interleukin-33 is preferentially expressed in ulceration-associated myofibroblasts. Am. J. Pathol..

[14] Seidelin JB (2010). IL-33 is upregulated in colonocytes of ulcerative colitis. Immunol. Lett..

[15] Kobori A (2010). Interleukin-33 expression is specifically enhanced in inflamed mucosa of ulcerative colitis. J. Gastroenterol..

[16] Gundersen MD (2016). Loss of interleukin 33 expression in colonic crypts—A potential marker for disease remission in ulcerative colitis. Sci. Rep..

[17] Sedhom MA (2013). Neutralisation of the interleukin-33/ST2 pathway ameliorates experimental colitis through enhancement of mucosal healing in mice. Gut.

[18] Moussion C, Ortega N, Girard JP (2008). The IL-1-like cytokine IL-33 is constitutively expressed in the nucleus of endothelial cells and epithelial cells in vivo: A novel 'alarmin'?. PLoS ONE.

[19] Dubois-Camacho K (2019). Inhibition of miR-378a-3p by inflammation enhances IL-33 levels: A novel mechanism of alarmin modulation in ulcerative colitis. Front. Immunol..

[20] Vertzoni M (2010). Characterization of the ascending colon fluids in ulcerative colitis. Pharm. Res..

[21] Schilli R (1982). Comparison of the composition of faecal fluid in Crohn's disease and ulcerative colitis. Gut.

[22] Delpire E, Gagnon KB (2018). Water homeostasis and cell volume maintenance and regulation. Curr. Top. Membr..

[23] Lee-Robichaud H, Thomas K, Morgan J, Nelson RL (2010). Lactulose versus polyethylene glycol for chronic constipation. Cochrane Database Syst. Rev..

[24] Nnane I (2020). The first-in-human study of CNTO 7160, an anti-interleukin-33 receptor monoclonal antibody, in healthy subjects and patients with asthma or atopic dermatitis. Br. J. Clin. Pharmacol..

[25] Swanson KD, Theodorou E, Kokkotou E (2018). Reproducing the human mucosal environment ex vivo: Inflammatory bowel disease as a paradigm. Curr. Opin. Gastroenterol..

[26] Powley IR (2020). Patient-derived explants (PDEs) as a powerful preclinical platform for anti-cancer drug and biomarker discovery. Br. J. Cancer.

[27] Russo I (2016). The culture of gut explants: A model to study the mucosal response. J. Immunol. Methods.

[28] Sundnes O (2015). Epidermal expression and regulation of interleukin-33 during homeostasis and inflammation: Strong species differences. J. Invest. Dermatol..

[29] Friedrich M, Pohin M, Powrie F (2019). Cytokine networks in the pathophysiology of inflammatory bowel disease. Immunity.

[30] Pietka W (2019). Hypo-osmotic stress drives IL-33 production in human keratinocytes—An epidermal homeostatic response. J. Invest. Dermatol..

[31] Shan J (2016). Interferon γ-induced nuclear interleukin-33 potentiates the release of esophageal epithelial derived cytokines. PLoS ONE.

[32] Schiering C (2014). The alarmin IL-33 promotes regulatory T-cell function in the intestine. Nature.

[33] Kuchler AM (2008). Nuclear interleukin-33 is generally expressed in resting endothelium but rapidly lost upon angiogenic or proinflammatory activation. Am. J. Pathol..

[34] Planell N (2013). Transcriptional analysis of the intestinal mucosa of patients with ulcerative colitis in remission reveals lasting epithelial cell alterations. Gut.

[35] Grauso M, Lan A, Andriamihaja M, Bouillaud F, Blachier F (2019). Hyperosmolar environment and intestinal epithelial cells: Impact on mitochondrial oxygen consumption, proliferation, and barrier function in vitro. Sci. Rep..

[36] Ip WKE, Medzhitov R (2015). Macrophages monitor tissue osmolarity and induce inflammatory response through NLRP3 and NLRC4 inflammasome activation. Nat. Commun..

[37] Hubert A, Cauliez B, Chedeville A, Husson A, Lavoinne A (2004). Osmotic stress, a proinflammatory signal in Caco-2 cells. Biochimie.

[38] Chovatiya R, Medzhitov R (2014). Stress, inflammation, and defense of homeostasis. Mol. Cell.

[39] Tropini C (2018). Transient osmotic perturbation causes long-term alteration to the gut microbiota. Cell.

[40] Brocker C, Thompson DC, Vasiliou V (2012). The role of hyperosmotic stress in inflammation and disease. Biomol. Concepts.

[41] Thrane AS (2011). Critical role of aquaporin-4 (AQP4) in astrocytic Ca^2+^ signaling events elicited by cerebral edema. Proc. Natl. Acad. Sci. USA.

[42] Rizopoulos T, Papadaki-Petrou H, Assimakopoulou M (2018). Expression profiling of the transient receptor potential vanilloid (TRPV) channels 1, 2, 3 and 4 in mucosal epithelium of human ulcerative colitis. Cells.

[43] Odenwald MA, Turner JR (2017). The intestinal epithelial barrier: A therapeutic target?. Nat. Rev. Gastroenterol. Hepatol..

[44] Ricanek P (2015). Reduced expression of aquaporins in human intestinal mucosa in early stage inflammatory bowel disease. Clin. Exp. Gastroenterol..

[45] Chen Y, Mu J, Zhu M, Mukherjee A, Zhang H (2020). Transient receptor potential channels and inflammatory bowel disease. Front. Immunol..

[46] Neurath MF (2014). Cytokines in inflammatory bowel disease. Nat. Rev. Immunol..

[47] Perez F (2020). IL-33 alarmin and its active proinflammatory fragments are released in small intestine in celiac disease. Front. Immunol..

[48] Krzystek-Korpacka M, Kempiński R, Bromke MA, Neubauer K (2020). Oxidative stress markers in inflammatory bowel diseases: Systematic review. Diagnostics (Basel).

[49] Latella G (2014). Results of the 4th scientific workshop of the ECCO (I): Pathophysiology of intestinal fibrosis in IBD. J. Crohns Colitis.

[50] Lopetuso LR (2018). IL-33 promotes recovery from acute colitis by inducing miR-320 to stimulate epithelial restitution and repair. Proc. Natl. Acad. Sci. USA.

[51] Of men, not mice. *Nat. Med.* **19**, 379. 10.1038/nm.3163 (2013). 10.1038/nm.3163 23558605

[52] Florholmen JR (2020). Discovery and validation of mucosal TNF expression combined with histological score—A biomarker for personalized treatment in ulcerative colitis. BMC Gastroenterol..

[53] Magro F (2017). Third European evidence-based consensus on diagnosis and management of ulcerative colitis. Part 1: Definitions, diagnosis, extra-intestinal manifestations, pregnancy, cancer surveillance, surgery, and ileo-anal pouch disorders. J. Crohns Colitis..

[54] Schroeder KW, Tremaine WJ, Ilstrup DM (1987). Coated oral 5-aminosalicylic acid therapy for mildly to moderately active ulcerative colitis. A randomized study. N. Engl. J. Med..

[55] Vadstrup K (2016). Validation and optimization of an ex vivo assay of intestinal mucosal biopsies in Crohn's disease: Reflects inflammation and drug effects. PLoS ONE.

[56] Fletcher PS (2006). Ex vivo culture of human colorectal tissue for the evaluation of candidate microbicides. AIDS.

[57] Browning TH, Trier JS (1969). Organ culture of mucosal biopsies of human small intestine. J. Clin. Investig..

[58] Gundersen MD (2019). Fibrosis mediators in the colonic mucosa of acute and healed ulcerative colitis. Clin. Transl. Gastroenterol..

[59] Pfaffl MW (2001). A new mathematical model for relative quantification in real-time RT–PCR. Nucleic Acids Res..

[60] Bankhead P (2017). QuPath: Open source software for digital pathology image analysis. Sci. Rep..

