# Supplementary material for: Hypo-osmotic stress induces the epithelial alarmin IL-33 in the colonic barrier of ulcerative colitis
Source: Sci Rep. 2022 Jul 7;12:11550. doi: 10.1038/s41598-022-15573-0 (PMC9263100; doi:10.1038/s41598-022-15573-0)
Supplement: Supplementary file 1 — Supplementary Information. [file 41598_2022_15573_MOESM1_ESM.pdf]

Supplementary files

Title

Hypo-osmotic stress induces the epithelial alarmin IL-33 in the colonic barrier of ulcerative colitis

Authors

Mona Dixon Gundersen*[1], Kenneth Bowitz Larsen[2], Kay Martin Johnsen [1,3], Rasmus Goll[1,3], Jon Florholmen[1], Guttorm Haraldsen[4]

Supplementary Table S1. Primary antibodies

**Specificity**	**Designation**	**Specification**	**Working concentration**	**Source**
human pSTAT3	(Tyr705)(D3A7) XP	rabbit monoclonal	1/400	CST
human pSTAT1	(Tyr701)(58D6)	rabbit monoclonal	1/400	CST
human IL33	Nessy-1	mouse monoclonal (IgG1)	1/1000	Enzo Life sciences
human Ki67	D2H10	rabbit monoclonal	1/400	CST
human CD34	HPA036722	rabbit polyclonal	1/400	Atlas antibodies
isotype control	DA1E XP	rabbit monoclonal IgG	Concentration matched	CST
isotype control	G3A1	mouse monoclonal IgG1	Concentration matched	CST

Abbreviations: CST (Cell signaling technologies)

Supplemental Figure S1. Dual staining of healthy controls

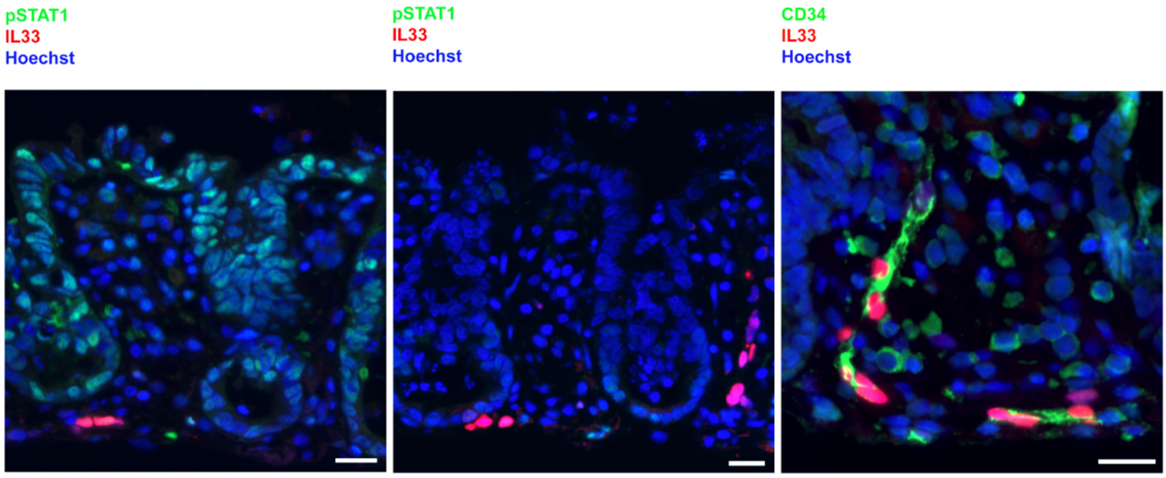

Panel shows immunofluorescence images for healthy controls. Image to the left shows a colonic biopsy stimulated for 24 hours with IFNG 100ng/ml. Image in the middle is a control biopsy without stimulant added to the basal medium for 24 hours where no pSTAT1 is seen in the epithelium. Image to the right shows a healthy control with dual staining with positive cells IL-33 (red) were found localised in the lamina propria CD34 positive vessels (green). Scale bars are given for 20 µm.
